# The impact of the coronavirus pandemic on the management of cancer patients in Lebanon: a single institutional experience

**DOI:** 10.2217/fon-2020-0313

**Published:** 2020-04-23

**Authors:** Clarisse Kattan, Hassan Badreddine, Elie Rassy, Hampig Raphael Kourie, Joseph Kattan

**Affiliations:** 1 Department of Hematology-Oncology, Hotel-Dieu de France University Hospital, Faculty of Medicine, Saint Joseph University, Beirut, Lebanon

**Keywords:** cancer patients, coronavirus, COVID-19, Lebanon, middle-income

The COVID-19 virus has started its spread in Wuhan, China back in December 2019 and evolved into a global pandemic surpassing all national barriers within weeks. This pandemic had social and economic implications on a worldwide scale and rapidly became an international public health crisis [1]. COVID-19 has a transmission rate ranging between 2 and 2.5 exceeding that of the seasonal flu (transmission rate of 1.3), Influenza A virus subtype H1N1 (transmission rate of 1.2–1.6) and Ebola (transmission rate of 1.6–2), but less than Severe Acute Respiratory Syndrome (SARS; transmission rate of 4) and Middle East Respiratory Syndrome (MERS; transmission rate of 2.5–7.2) [2]. The WHO declared COVID-19 a worldwide pandemic disease on 12 March 2020 and urged countries to limit its spread [3]. In the absence of a proven effective antiviral agent, social distancing is the primary intervention and self-initiated quarantine for 14 days by people with respiratory symptoms to hinder the spread of the viral infection [4,5]. Nevertheless, the COVID-19 virus continued its worldwide spread leading to many epicenters across Europe and the USA [6].

The implications of the pandemic on middle and low-income countries have not been fully reported. Hopman *et al.* considered that low-to-middle-income countries will face many difficulties as for the containment of the pandemic when compared with high-income countries such as China [7]. Essential requirement for handling a pandemic include: isolation, quarantine, social distancing, community containment measures and curative treatment (if available). However, low-to-middle-income countries are not fully prepared to afford large-scale diagnostics and are not equipped with ICU beds and personnel trained in critical care [7]. Lebanon is a high-middle income Middle-Eastern country according to the World Bank; but has been grappling over the last months with its worst economic turmoil since the 1975–1990 civil war [8]. The population in Lebanon is estimated at around 4.8 million, relatively elder population with 12.4% of the habitants older than 60 years of age, served by 14,864 hospital beds, and having a high life expectancy age 80 years [9]. By early April 2020, around 500 COVID-19 patients were identified among 6500 people tested with clinical symptoms or history of exposition to the virus. There were 17 related deaths during this period including two patients with advanced lung cancer and renal cell carcinoma each [10].

## Our guidelines in the management of patients with cancer during the COVID-19 pandemic

Epidemiology data reports that the number of infected patients has surpassed 1 million cases and killed more than 70,000 [6]. The first Chinese nationwide study by Liang *et al.* from the Chinese epicenter suggests that cancer patients have a higher risk of COVID-19 infection (1 vs 0.29%) and a higher risk of severe illness and a need for intensive care assistance (39 vs 8%; p = 0.0003) [11]. A history of anticancer treatment or surgery in the past month was associated with poor outcomes (OR = 4.079; 95% CI: 1.086–15.322). This susceptibility to severe infections was attributed to the immunosuppressive effect of the primary disease and anticancer treatments [11]. The provision of care promptly faces many difficulties since the exposition to a hospital setting in such times carries an increased risk for infection in these patients. Hospital admission and recurrent hospital visits were found to be a potential risk factor for COVID-19 infection (OR = 4.079; 95% CI: 1.086–15.322) [12]. Overall, cancer patients seem at a higher risk of severe events in 48–54% of cases and death in 5.6–29% [13–15].

In line with the major international societies, the Lebanese Society of Medical Oncology (LSMO) issued guidelines to help oncologists provide the optimal care for cancer patients while reducing their chances of contracting or transmitting the COVID-19 infection (Box 1). These guidelines mainly focused on the necessity to screen patients for possible COVID-19 infection to ensure their admission to the COVID-19 unit rather than the outpatient department or oncology floor, as well as deferring their anticancer treatment till full recovery. Indeed, chemotherapy has an immunosuppressive effect which puts the patients at risk of being infected and immune checkpoint inhibitors increase the risk of cytokine release syndrome if the patients are infected [16]. The LSMO guidelines stressed the necessity to prioritize patients on a case-by-case scenario favoring curative rather than palliative therapy, oral rather than intravenous treatment and recommending therapeutic pause if plausible [17] (Box 1). We have also published in the *Future Oncology* journal an overview of available evidence concerning COVID-19 in cancer patients to better guide management decisions [18].

Box 1.  **The recommendations of the Lebanese Society of Medical Oncology for managing cancer patients during the COVID-19 pandemic (modified).**

1. **Prevention of contamination:** Screening of patients and visitors for evocative symptoms notably respiratory symptoms and fever. Do not admit patients with confirmed COVID-19 infection or suspicious cases to the oncology departments and privilege their admission in COVID-19 dedicated departments for management.
2. **Prioritization** of patients by favoring curative therapies over palliative treatment plans, delaying or withholding anticancer treatment cycles when justified.
3. **Avoid overcrowded clinics** by favoring teleconsultation and modifying treatment regimens which lower the number of patients receiving weekly chemotherapy and privileging oral treatment when possible.
4. **Sanctuarization of oncology department:** Withhold anticancer treatment of infected patients with COVID-19 until full recovery. The admission of COVID-19 patients should be done in dedicated departments.
5. Manage patients in need of supportive care and palliation by teleconsultation and limiting hospital or clinic visits.

## Our experience in the management of patients with cancer during the COVID-19 pandemic

Hotel Dieu University Hospital is a 600-bed tertiary and referral hospital located in Beirut serving the Lebanese population. The Hematology-Oncology Department occupies two floors with a total of 50 beds serving around 20% of the Lebanese cancer patients. The medical team includes 9 senior oncologists, 7 senior oncology fellows, 5 interns, and 24 nurses and 16 practical nurses. As a result of the lockdown state imposed by the Lebanese Government, our head of department (coauthor of the LSMO guidelines and senior author [JK] of this manuscript) elected to abide by the LSMO guidelines in line with the majority of the oncology societies [19–23]. The application of these measures started at the beginning of March 2020 by contacting patients or family members to clearly explain these new measures. Patients were also screened for the presence of respiratory symptoms before presenting to the hospital. We noted that the patients and their family feared of contracting the COVID-19 infection during the hospital and were reluctant to accept any treatment modification or suspension except for few elderly patients that accepted the switch to oral chemotherapy such as replacing 5-fluorouracil with capecitabine for colon cancer patients and hormonal therapy for castrate resistance prostate cancer patients. The number of hospitalized patients at the 1-day unit during February, March and April 2020 remained comparable to the same period during the last year.

Besides, our institution imposed some measures at the same-day unit to minimize the risk of infection on patients who were admitted. These included screening patients for any respiratory symptoms or fever before the initiation of treatment, limiting the number of visitors to one person only during the hospitalization period, mandating mask protecting for both the patient and the personnel during. Radiotherapy indication and duration were discussed case-by-case. Surgeries were delayed for the localized tumors, and a special emphasis on neoadjuvant therapy was made until containment of the pandemic.

Our practice by phone screening patients for fever and respiratory symptoms has protected our patients as well as our medical team from any COVID-19 infection over the last 6 weeks (first COVID-19 confirmed case in Lebanon was identified on 21 February 2020). We encountered several limitations in applying our institutional and LSMO guidelines. Patients were reluctant to accept a therapeutic pause by fear of disease progression, more patientsspecifically patients undergoing palliative treatment refused any type of treatment modification. We have previously reported on this behavior among our cancer patients who often lean toward an oncologic treatment even during the last month of life [24].

## Conclusion

Lebanon is one of the most dynamic healthcare systems and a regional leader in healthcare among middle-income countries. Unfortunately, the healthcare system has been weakened under the pressure of the economic recession, unstable political climate, shortage in nurses and basic help staff. Our experience showed that cancer patients are aware of the COVID-19 morbidity and fear its complications. Nevertheless, we were not able to completely implement the recommendations suggested by the LSMO as well as international societies. Patients were very reluctant to delay or modify their treatment plan although it was medically reasonable. Our approach consisting of screening patients for signs of infections, limiting hospital visits, wearing protective masks by the medical team as well as the patients, postponing surgeries and limiting radiotherapy when possible, protected our patients and our medical team from COVID-19 infection.
